# Social norms and group-bounded indirect reciprocity

**DOI:** 10.1017/ehs.2026.10045

**Published:** 2026-03-31

**Authors:** Wakaba Tateishi, Hirotaka Imada

**Affiliations:** 1 Department of Business Administration, Hokkaido Musashi Women’s University, Sapporo, Japan; 2 Department of Psychology, Royal Holloway, University of London, Egham, UK; 3 School of Economics and Management, Kochi University of Technology, Kochi, Japan

**Keywords:** indirect reciprocity, cooperation, in-group favouritism, reputation, intergroup cooperation

## Abstract

Indirect reciprocity is a reputation-based mechanism proposed to explain the evolution of human cooperation. Theoretical models demonstrated that the use of both first-order information (i.e., whether an evaluation target cooperated) and second-order information (i.e., the reputation of an interaction partner of the evaluation target) is critical for the evolution of cooperation. However, empirical findings on the use of second-order information have been mixed. Drawing upon the literature on group-bounded indirect reciprocity, we tested the hypothesis that individuals would be more sensitive to second-order information when evaluating in-group interactions, compared to when evaluating out-group interactions. We conducted a preregistered online experiment (*N* = 604), where we independently manipulated group membership (in-group vs. out-group), target behaviour (cooperation vs. defection), and recipient reputation (good vs. bad). We found that donors who defected against good recipients were rated more negatively than those who defected against bad recipients, indicating the use of second-order information. Partly consistently with our hypothesis, when individuals evaluated coopering donors, second-order information influenced reputation for in-group donor–recipient interactions more than for out-group donor–recipient interactions. Nevertheless, individuals readily used second-order information, whether or not they evaluated in-group or out-group donor–recipient interactions.

## Graphical abstract

**Figure:**
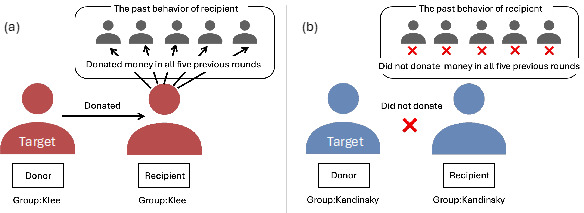

## Social Media Summary

An experiment found that second-order information influenced reputation of cooperators more within the group boundary than across it.

## 1. Introduction

Humans are a cooperative species, displaying cooperation even with unrelated individuals (Bowles & Gintis, 2011; Fehr & Fischbacher, 2003). Cooperation refers to behaviours that serve to maximise collective benefits rather than individual benefits (Van Lange et al., 2013). The personally costly nature of cooperation set up a theoretical conundrum against the prevalence of cooperation: why do people cooperate, and why has cooperation evolved? One of the prominent theories to explain the evolution of cooperation is indirect reciprocity (Rand & Nowak, 2013). Indirect reciprocity is a system in which participating members cooperate only with others with a good reputation (Alexander, 1987; Nowak & Sigmund, 1998a, 1998b). Indirect reciprocity can be described as ‘my actions toward you depend on your previous behaviour toward others.’ Indirect reciprocity has been shown to maintain cooperation through theoretical studies (Ohtsuki & Iwasa, 2006; Panchanathan & Boyd, 2003), even when individuals only privately assess each other (Fujimoto & Ohtsuki, 2023). Experimental studies and field studies also demonstrated that the indirect reciprocity works to maintain cooperation in human societies (Dores Cruz et al., 2021; Engelmann & Fischbacher, 2009; Milinski, 2016; Wu et al., 2021). Importantly, though, for indirect reciprocity to sustain cooperation, past theoretical work suggests it is imperative that individuals confer reputation to others based on the second-order information (i.e., whether others cooperated with or defected against a person with a good or bad reputation). Nevertheless, the previous empirical literature has produced mixed evidence, posing a challenge to the application of indirect reciprocity to understand human cooperation. To fill the gap, drawing upon the literature on group-bounded indirect reciprocity (GBIR), we examined the role of group membership as a boundary condition for the use of second-order information.

### 1.1. First-order and second-order information in indirect reciprocity

Studies using mathematical models revealed the conditions in which cooperation evolves under indirect reciprocity; Nowak and Sigmund (1998b) proposed a model of indirect reciprocity by describing the evolution of discriminators who decide whether to cooperate based on others’ reputations. In their model, they set two roles: a donor and a recipient. A donor decided whether to cooperate with a recipient by incurring a cost *c* to provide a benefit *b* to a recipient (*c* < *b*). They proposed the simplest discriminator strategy in indirect reciprocity, called the image-scoring strategy. This strategy uses information about whether a donor cooperated or not in the past to determine the donor’s reputation, regardless of whether their recipient had a positive or negative reputation. In other words, this image-scoring strategy only utilised first-order information. Subsequent theoretical studies have raised doubts about the image-scoring strategy, revealing that it does not allow cooperation to be stabilised when errors, such as those included in executing intended cooperation, are introduced (e.g., Leimar & Hammerstein, 2001; Panchanathan & Boyd, 2003).

Ohtsuki and Iwasa (2004) systematically examined the condition in which cooperation by indirect reciprocity evolves despite the occurrence of errors, and identified eight social norms, the ‘leading eight’. Social norms refer to rules for assigning reputations to donors; for example, image-scoring is one of these social norms. It includes the standing norm (Leimar & Hammerstein, 2001; Panchanathan & Boyd, 2003; Sugden, 1986), which regards the donor’s defection against a bad recipient as good (i.e., justified defection). Ohtsuki and Iwasa (2004) highlighted the importance of using not only first-order information (i.e., information about the donor’s past behaviour) but also second-order information (i.e., information about whether the recipient of that behaviour had a good or bad reputation); the leading eight norms share two crucial characteristics. First, cooperation with good persons is regarded as good while defection against them is regarded as bad. Second, defection against bad persons is regarded as a good behaviour, which means that a justified defection acquires a good reputation. The use of second-order information is essential for the evolution of indirect reciprocity and is critically important in evolutionary simulations and mathematical model studies (Ohtsuki & Iwasa, 2006; Pacheco et al., 2006; Panchanathan & Boyd, 2003; Takahashi & Mashima, 2006).

### 1.2. Empirical studies of the use of first-order and second-order information

Despite the critical role of the use of second-order information in the evolution of cooperation under indirect reciprocity, the current literature lacks robust experimental evidence. Specifically, while previous work has consistently documented the use of first-order information (Engelmann & Fischbacher, 2009; Seinen & Schram, 2006; Wedekind & Milinski, 2000), previous experimental studies have yielded evidence in favour of and against the use of the second-order information (Bolton et al., 2005; Mashima & Takahashi, 2008; Milinski et al., 2001; Okada et al., 2018; Swakman et al., 2016; Ule et al., 2009; Yamamoto et al., 2020).

Milinski et al. (2001) empirically investigated whether humans rely solely on first-order information or also use second-order information when deciding whether to cooperate with others. Participants were assigned the role of either a donor or a recipient in each round. Donors decided whether to cooperate with a recipient. Milinski et al. (2001) compared the probability of donors who cooperated or defected with a defector in two conditions: the low-information condition (first-order information), where only the donor’s past behaviour was visible, and the high-information condition (second-order information), where both the donor’s and their recipients’ past behaviours were displayed. The results showed no difference between the two conditions, which implies that people do not use second-order information.

Ule et al. (2009) demonstrated that while some people used both first-order and second-order information, the majority relied only on first-order information. Donors were paired with recipients and could choose to help (incurring a cost to themselves but benefiting the recipient: cooperate), pass (do nothing: defect), or punish (incurring a cost to themselves to harm the recipient). Donors were provided with first-order information and had the option to obtain second-order information at a small cost. Ule et al. (2009) examined the distribution of participants’ strategies and found that participants who conditionally cooperated relied solely on first-order information about twice as often as those who used both first-order and second-order information. Together with Milinski et al. (2001), Ule et al. (2009) suggest that the use of second-order information does not play a critical role in actual cooperation decision-making.

Other studies have reported that people did not use second-order information when the donor’s behaviour (i.e., first-order information) was cooperation, but tended to use second-order information when the donor’s behaviour was defection (Okada et al., 2018; Swakman et al., 2016; Yamamoto et al., 2020). Swakman et al. (2016) examined whether people would access second-order information even at a cost and whether they used first-order and second-order information when making cooperation decisions. The results showed that participants used second-order information when the donor’s behaviour was defection, and they were more likely to cooperate with donors who defected against a bad recipient than those who defected against a good recipient. In addition, Okada et al. (2018) examined how people disclose first-order and second-order information and whether they use these types of information in their cooperation decisions. Their findings indicated that while the majority of individuals disclosed both types of information, first-order information was prioritised. Furthermore, when the first-order information indicated cooperation, individuals cooperated regardless of the second-order information. Finally, Yamamoto et al. (2020) conducted vignette studies to examine how people evaluate a donor when both first-order and second-order information are provided. The results showed that cooperation with both good and bad recipients was evaluated positively, while defection against good recipients was evaluated negatively. However, defection against bad recipients received neutral evaluations. Thus, second-order information affected the evaluation of the donor, at least, when the donor’s behaviour was defection.

On the other hand, Mashima and Takahashi (2008) suggested that people utilise second-order information even when a donor’s past behaviour (i.e., first-order information) was ‘cooperate’. They examined the evaluation of donors when both first-order and second-order information were provided through a vignette study. Similar to Okada et al. (2018), Swakman et al. (2016), and Yamamoto et al. (2020), justified defection was evaluated positively. While cooperation with a bad recipient was perceived as more generous than cooperation with a good recipient, cooperation with a good recipient was rated higher in terms of social appropriateness, social order, expectation of positive treatment by others, and willingness to cooperate with the donor. Unlike previous studies, their findings demonstrated that people utilise second-order information in both cases where the first-order information indicates cooperation and where it indicates defection.

In addition, Bolton et al. (2005) suggested that the use of second-order information promotes cooperation. In their study, there were two roles: donor and recipient, and the donor had to decide whether to cooperate with the recipient. Three conditions were tested: the donor was either given no information, first-order information (i.e., the recipient’s past behaviour when the recipient role was donor), or both first-order and second-order information (i.e., the recipient’s previous recipient’s past behaviour). The results showed that cooperation rates were highest when second-order information was provided. Looking at the breakdown of cooperation rates, donors who cooperated with good recipients were more likely to be cooperated with than donors who cooperated with bad recipients. Similarly, when the first-order action was defection, donors who defected against bad recipients were more likely to be cooperated with than donors who defected against good recipients. As reviewed in Appendix A, the previous studies substantially vary in experimental settings, and it is hard to systematically draw conclusions as to what contributed to the mixed evidence in the literature (see Appendix A for a review of relevant previous empirical studies).

### 1.3. Group-bounded indirect reciprocity

Milinski et al. (2001) found evidence that individuals are indifferent to second-order information and assign good and bad reputations to those who cooperated and defected, respectively. They pointed out that social norms that require the use of second-order information might be too demanding due to limited cognitive and memory capacity. When individuals employ a social norm that involves the use of second-order information, they need to remember and attend to the history of past social interactions involving many different individuals. It is thus conceivable that despite that social norms that require second-order information are not viable for humans despite that theoretical work consistently points to the importance of the use of second-order information in the evolution of cooperation under indirect reciprocity. In the present research, we propose and test the possibility that the assumption that indirect reciprocity is bounded by group membership reduces the cognitive load, and allows the use of second-order information by limiting the use of second-order information only for within-group interactions. Previous studies on GBIR (e.g., Imada et al., 2023a, 2024a), in fact, suggest that individuals have such an assumption and the assumption guides intergroup cooperation.

According to GBIR (Imada, Mifune, & Shimizu, 2024a; Imada, Romano, et al., 2023b; Mifune et al., 2010; Yamagishi et al., 1999), shared group membership is a critical determinant of the perceived realm of indirect reciprocity. Previous studies have suggested that in-group membership, by default, functions as a cue of indirect reciprocity and thus individuals intuitively assume that in-group members, but not necessarily out-group members, belong to the same system of indirect reciprocity (Imada, Mifune, & Shimizu, 2024a; Imada, Mifune, & Zibell, 2024b; Imada, Romano, et al., 2023b; Yamagishi et al., 1999; Yamagishi & Kiyonari, 2000; Yamagishi & Mifune, 2008; Yamagishi et al., 2008). Consequently, individuals can expect in-group members to be cooperative with them (the expectation hypothesis: Imada et al., 2023a; Imada, Mifune, & Shimizu, 2024a; Yamagishi et al., 1999, 2008) and they experience an increased level of reputational concern in the eyes of in-group members (the reputation management hypothesis: Kajiwara et al., 2022; Mifune et al., 2010; Mifune & Yamagishi, 2015; Yamagishi & Mifune, 2008; but also see Horita & Hamada, 2024). These psychological processes, in turn, lead to increased cooperation with in-group members, i.e., in-group favouritism (Balliet et al., 2014; Imada, Mifune, & Shimizu, 2024a).

Given the experimental evidence of cooperation guided by GBIR, we propose that group membership influences whether individuals use second-order information to evaluate others. More specifically, we hypothesised that when individuals evaluate an in-group donor’s behaviour towards an in-group recipient, they should consider whether the in-group recipient has a positive or negative reputation. Contrastingly, when observing a donor–recipient interaction between out-group members, since this interaction should be perceived to take place outside of the relevant reputation system, people would not be accustomed to use second-order information.

### 1.4. The present study

We conducted a highly powered online experiment to test the preregistered hypothesis that individuals are more sensitive to second-order information (i.e., whether a recipient has a good or bad reputation) when assigning reputation, particularly when both the donor and the recipient belong to their own group, compared to when both belong to an out-group. More specifically, we asked participants to evaluate donors in 16 different scenarios, in which we independently manipulated (a) the group membership of the donor (in-group vs. out-group), (b) the group membership of the recipient (in-group vs. out-group), (c) the donor’s behaviour (cooperation vs. defection), and (d) the recipient’s reputation (good vs. bad). Participants rated the reputation of each donor as a target on a scale from 0 (very bad) to 100 (very good). The primary aim of our study was to disentangle the mixed empirical evidence regarding the use of second-order information by testing the role of group membership.

Our study would also contribute to the empirical literature on GBIR and cooperation; as discussed earlier, GBIR has been a guiding perspective to explain why individuals display in-group favouritism (Balliet et al., 2014; Everett et al., 2015; Imada, Mifune, & Shimizu, 2024a; Yamagishi et al., 1999). Nevertheless, while previous studies consistently supported the expectation hypothesis, they have found mixed evidence for the reputation management hypothesis. Supporting the hypothesis, Mifune et al. (2010) found that an image of watching eyes, which was designed to trigger reputational concern, promoted prosocial behaviour towards in-group members but not towards out-group members. Two studies found a correlation between trait reputational concern and in-group favouritism (Kajiwara et al., 2022; Mifune & Yamagishi, 2015). Contrastingly, however, a recent study failed to replicate the correlation between trait reputational concern and in-group favouritism (Horita & Hamada, 2024) and several experiments suggest that group membership does not moderate the cooperation-enhancing effect of reputational concern (Imada et al., 2023a; Imada, Mifune, & Shimizu, 2024a; Imada, Romano, et al., 2023b; Romano et al., 2017). Imada, Mifune & Zibell (2024b) further found that individuals expect both in-group and out-group members to be willing to gossip about their behaviour towards in-group members, suggesting that they should be worried about how they are perceived equally by in-group and out-group members. In sum, those previous studies offered evidence against the reputation management theory in terms of the cooperation-enhancing effect of reputation and the use of reputation. By investigating whether social norms are group-bounded, our study can test the reputation management hypotheses from a new angle: reputation assignment mechanisms.

Prior to data collection, we preregistered the target sample size, data exclusion criteria, hypotheses, and analytic approaches (https://osf.io/b5akc/overview). We have study material, data, analysis codes, and supplementary materials available at https://osf.io/mkh57/overview.

## 2. Method

### 2.1. Participants and design

The study followed a 2 (donor group: in-group vs. out-group) × 2 (recipient group: in-group vs. out-group) × 2 (donor behaviour: cooperate vs. defect) × 2 (recipient reputation: good vs. bad) within-subjects design. We recruited and paid 600 Japanese participants via Lancers (https://www.lancers.jp/). There were four individuals who fully completed the study without properly signing up for it. Consequently, we had 604 complete, non-duplicate responses (364 men, 238 women, 2 other, *M*_age_ = 44.17, *SD*_age_ = 9.85). Our target sample size was determined on the basis of our budgetary constraints. This study was approved by [redacted for anonymous peer review].

### 2.2. Procedure

After giving consent, participants were presented with 13 pairs of paintings, one by Paul Klee and the other by Wassily Kandinsky, and they indicated which one they preferred. We informed participants that they were either a member of Klee or Kandinsky group, based on their actual responses. We introduced this task to establish arbitrarily created experimental groups (i.e., minimal group paradigm: Rabbie & Horwitz, 1969; Tajfel et al., 1971).

**Figure 1.**
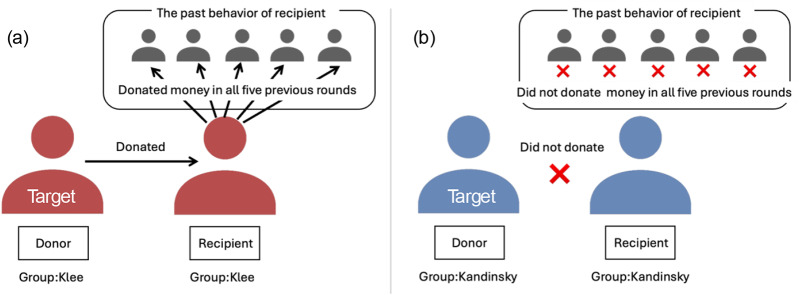
Experimental instruction.

*Note*: The figure illustrates 2 scenarios out of the 16 scenarios: (a) A scenario where the donor (Klee group) donated to the recipient (Klee group) with a good reputation: (b) A scenario where the donor (Kandinsky) did not donate to the recipient (Kandinsky) with a bad reputation.

Participants were then given instructions for a giving game. There were two roles in the giving game: donor and recipient. A donor and a recipient are randomly matched in an iterated game. The donor decides whether to donate (cooperate) their endowment to the recipient or not (defect). The money transferred to their recipients is tripled before it is given to the recipient. Donors are given information about the past behaviours of their recipient when making their decision; whether the recipient had donated their endowment or not in the previous five rounds when the recipient played the role of donor. After reading the instructions, participants answered four comprehension check questions. They were given a correct answer and further instructions for questions they incorrectly answered. We created the task based on the experimental vignette developed and used by Yamamoto et al. (2020). Following Yamamoto et al. (2020), we used the term ‘sender’ instead of ‘donor’ in the instructions and descriptions to facilitate the understanding of the scenario (see Fig. 1).

Participants were then presented with 16 different scenarios varying in the group membership of the donor and the recipient as well as the donor’s behaviour (donated vs. did not donate) and the recipient’s reputation (good: donated in the last five rounds as a donor; bad: did not donate in the last five rounds as a donor), in a randomized order (Fig. 1). Participants evaluated 16 different donors as targets. First, they rated the reputation of each donor on a scale from 0 (bad) to 100 (good). In addition to the preregistered reputation ratings, the following exploratory measures were included. To measure their willingness to donate to the donors, participants indicated whether they would donate or not donate to the donors if paired with them (i.e., participants as the donor and the donor as the recipient) in the giving game. Participants also rated their impressions of the targets on five dimensions: warmth, trustworthiness, generosity, competence, and likability, using a scale from 1 (does not describe the target at all) to 7 (describes the target extremely well). We note that before participants completed those measures, they were presented with four comprehension check questions about the manipulated information (whether the donor belonged to the same or different group, whether the receiver belonged to the same or different group, whether the donor donated or not, and whether the receiver had donated in the past trials). If they did not correctly answer the questions, they were presented with correct information and proceeded only after selecting the correct answer. This ensured that participants understood the experimental condition. After completing these tasks, we collected demographic information (sex and age).

## 3. Results

### 3.1. Reputation

In the preregistration, we planned to conduct a 2 (donor group: in-group vs. out-group) × 2 (recipient group: in-group vs. out-group) × 2 (donor behaviour: cooperate vs. defect) × 2 (recipient reputation: good vs. bad) within-subjects ANOVA and follow up significant interactions with simple main effect analyses. However, we deviated from our preregistration in two major ways: first, we adopted a Bayesian estimation, which allowed us to test the presence and absence of effects. Second, we created hypothesis-relevant dummy coding to reduce the complexity of the model and improve the interpretability and clarity of hypothesis testing.

We hypothesised that participants would be more sensitive to second-order information (i.e., whether a recipient had a good or bad reputation) when both the donor and the recipient belonged to the in-group, compared to when they both belonged to the out-group. To examine this hypothesis, we examined whether the difference in donors’ reputation scores between good- and bad-reputation recipients was larger when both donor and recipient belonged to the in-group than when both belonged to the out-group, separately for cases in which the donor cooperated and defected.

We constructed a regression model using dummy variables (Eq. 1). To represent the dummy variables for each experimental condition, we employ Iverson brackets, where [*P*] denotes a value of 1 if the logical proposition *P* is true and 0 otherwise. In this model, dummy variables were set based on the group membership of the donor (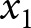), the group membership of the recipient (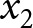), the donor’s behaviour (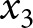), and the recipient’s reputation (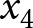). The reference category was defined as the condition in which the donor belonged to the in-group (
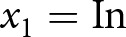
), the recipient belonged to the in-group (
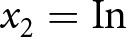
), the donor cooperated (
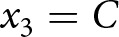
), and the recipient had a bad reputation (
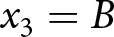
).
(1)
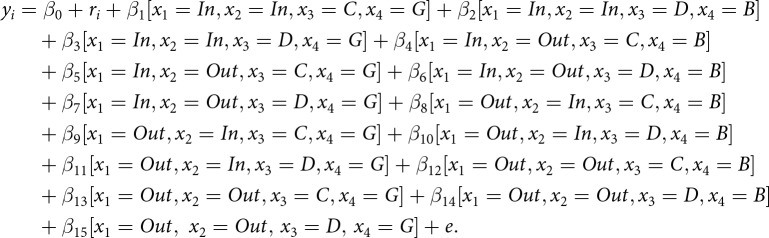


If the donor cooperates, we expect the donor’s reputation score to be higher when the recipient has a good reputation than when the recipient has a bad reputation. In addition, this difference should be larger when the donor and recipient belong to the in-group than when they belong to the out-group. This can be formally expressed by the following inequality (Eq. 2):
(2)
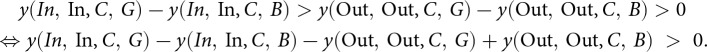


We rewrote the left-hand side of Eq. 2 as a single linear contrast, 
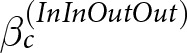
, by substituting the corresponding parameters from Eq. 1. We then estimated the posterior distribution of this contrast using the Bayesian model. Again, the condition in which the donor and recipient belong to the in-group, the donor cooperates, and the recipient has a bad reputation (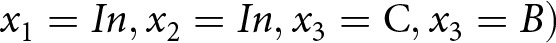 was the reference category. 
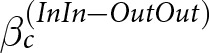
 captures the difference between in-group and out-group donor–recipient pairs in the change in the donor’s reputation score associated with the recipient’s reputation (good vs. bad), conditional on donor cooperation. We interpreted the results based on the posterior mean of 
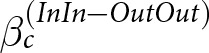
. A positive value of 
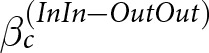
 indicates that the difference in the reputation score (good vs. bad recipient) is larger for in-group pairs than for out-group pairs, thereby supporting our hypothesis. The value of 
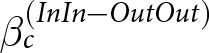
 close to zero indicates that this difference does not vary by group membership. In contrast, a negative value of 
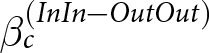
 indicates that the difference in the reputation score is larger for out-group pairs than for in-group pairs.
(3)
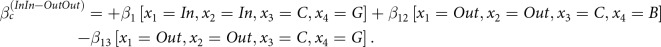


If the donor defects, we expect the donor’s reputation score to be lower when the recipient has a good reputation than when the recipient has a bad reputation. In addition, this difference should be larger when the donor and recipient belong to the in-group than when they belong to the out-group. Thus, our hypothesis for when the donor defects can be formally expressed as the following (Eq. 4):
(4)
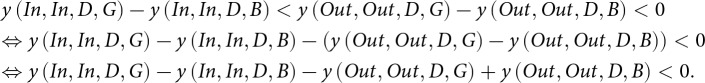


We rewrote the left-hand side of Eq. 4 as a single linear contrast, 
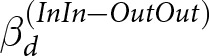
, by substituting the corresponding parameters from Eq. 1. We then estimated the posterior distribution of this contrast using the Bayesian model. We again expressed the equation as a single linear contrast by substituting the corresponding parameters from Eq. 1. This contrast is denoted as 
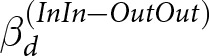
 and it captures the difference in reputation scores between in-group and out-group donor–recipient pairs associated with the recipient’s reputation (good vs. bad), conditional on donor defection. We interpreted the results based on the estimated mean of 
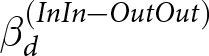
. A negative value of 
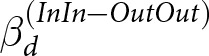
indicates that the difference in the reputation score is larger for in-group pairs than for out-group pairs, thereby supporting our hypothesis. A value of 
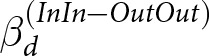
close to zero indicates that this difference does not vary by group membership. In contrast, a positive value of indicates that the difference in the reputation score is larger for out-group pairs than for in-group pairs.
(5)
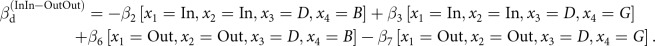


**Figure 2.**
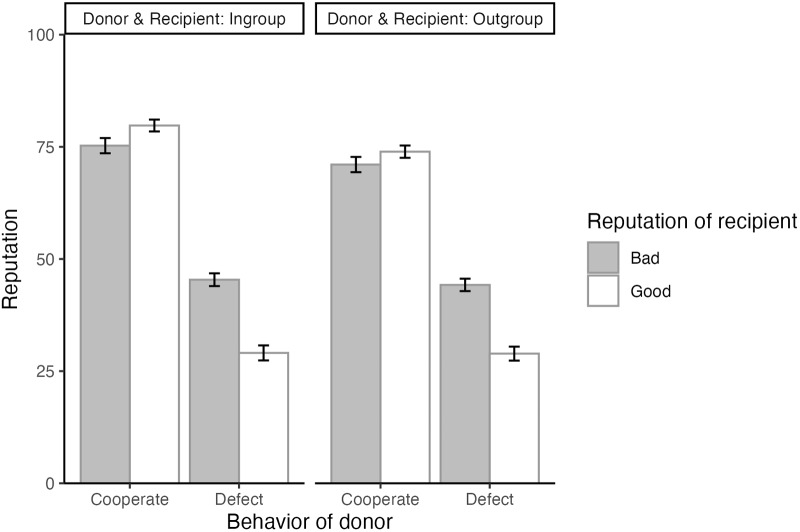
Donor’s reputation scores (Bad: 0 to Good: 100) by donor behaviour, recipient’s reputation, and group membership.

Figure 2 presents the mean of the donor’s reputation across conditions. We estimated the parameters of 
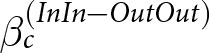
 and 
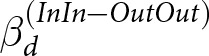
by using brms (Bürkner, 2017), an R package that interfaces with probabilistic programming language STAN to estimate the posterior distribution using Markov Chain Monte Carlo (MCMC) algorithms. Models were fitted using weakly informative priors, Normal (0, 5) on beta coefficients, and Student’s *t* (3, 0, 2.5) on the standard deviation of varying effects (i.e., participants). The parameters were estimated using four MCMC chains, each with 2000 iterations and 1000 warm-ups. The convergence of the MCMC was confirmed. We interpreted the effect of 
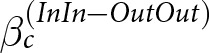
 and 
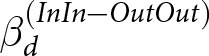
 based on the means and standard deviations of the estimates, as well as the widths of the Bayesian 95% credible intervals (CI).

We hypothesised that participants are sensitive to second-order information (i.e., whether a recipient has a good or bad reputation) when rating a donor, particularly when both the donor and the recipient belong to their in-group, compared to when they both belong to an out-group. The posterior estimate of 
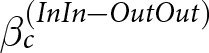
 was credibly positive, with the entire 95% credible interval in the positive range (*b* = 6.03, SD = 1.39, 95% CI [3.28, 8.76]). On the other hand, that of 
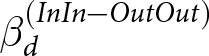
 was not credibly diferent from zero (*b* = −0.98, SD = 1.44, 95% CI [−3.78, 1.74]). These results provide partial support for our hypothesis. When the donor cooperated, the difference in donor evaluations based on second-order information (i.e., whether the recipient had a good or bad reputation) was larger for in-group donor–recipient pairs than for out-group pairs. In other words, participants were more sensitive to second-order information when both the donor and the recipient belonged to the in-group than when they belonged to the out-group, but only in the case of cooperative behaviour. By contrast, this pattern did not emerge when the donor defected, indicating that the heightened sensitivity to second-order information was observed only when the donor cooperated.

We further followed up the results by comparing whether the difference in donor evaluations based on second-order information across four group conditions when the donor cooperated: In–In: the donor and recipient both belonged to the in-group; Out–Out: the donor and recipient both belonged to the out-group; In–Out/Out–In: the donor and recipient belonged to the in-group and the out-group, respectively or vice versa. We found that the effect of second-order information was higher in the In–In condition than in the In–Out (*b* = 4.48, SD = 1.36, 95% CI [1.70, 7.20]) and Out–In (*b* = 7.79, SD = 1.34, 95% CI [5.29, 10.43]) conditions, and that in the Out–Out condition was not different from those in the In–Out (*b* = − 1.55, SD = 1.42, 95% CI [−4.28, 1.29]) and Out–In (*b* = 1.77, SD = 1.43, 95% CI [−1.11, 4.66]) conditions. Thus, when evaluating cooperators, the effect of second-order information was stronger only if the donor and the recipient both belonged to the in-group. This suggests that people are more sensitive to second-order information when evaluating within-group interactions, supporting the idea of GBIR.

**Figure 3.**
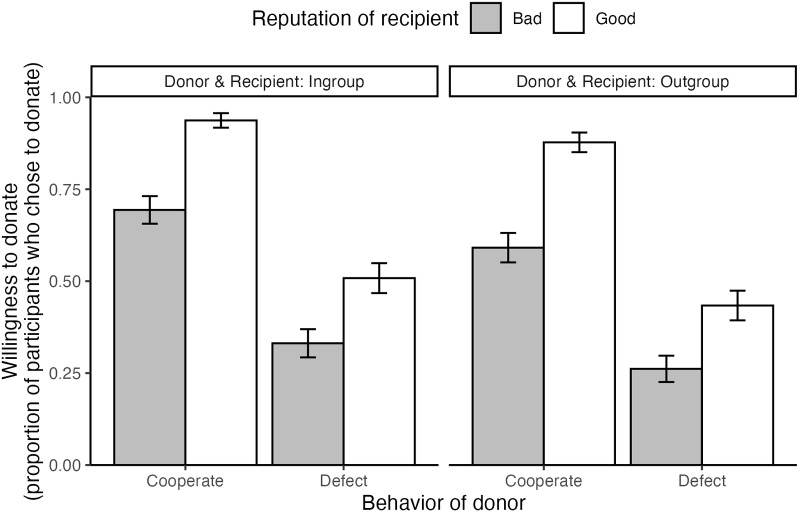
The willingness to donate (donated or did not donate) to the donor, by donor behaviour, recipient’s reputation, and group membership.

As an exploratory analysis, we conducted a 2 (donor and recipient’s group: in-group vs. out-group) × 2 (donor’s behaviour: cooperate vs. defect) × 2 (recipient’s reputation: good vs. bad) generalized linear mixed model analysis with a random intercept (participant’s ID) on the donor’s reputation (Fig. 3). We constructed a regression model as follows (Eq. 6):
(6)
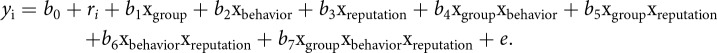

**Figure 4.**
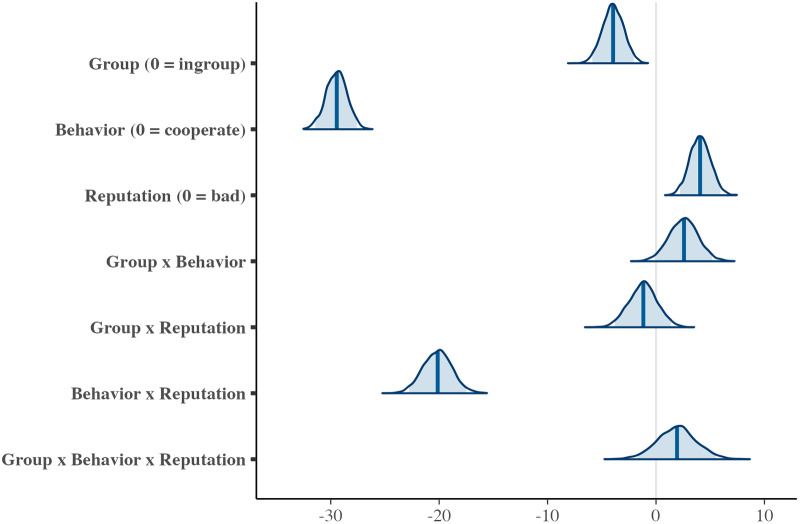
The estimated result of the score of reputation.

*Note*: The figure shows posterior distributions of the estimated parameters. The solid vertical line represents the posterior mean, and the shaded blue areas indicate the 95% Bayesian credible intervals.

This model was analysed using {brms} (Bürkner, 2017). Models were fitted using weakly informative priors, Normal (0, 5) on beta coefficients, and Student’s *t* (3, 0, 2.5) on the standard deviation of varying effects (i.e., participants). The parameters were estimated using four MCMC chains, each with 2000 iterations and 1000 warm-ups. The convergence of the MCMC was confirmed. We interpreted the effect of each factor based on the means and standard deviations of the estimates, as well as the widths of the Bayesian 95% credible intervals (estimated results: Fig. 4).

The interaction effect of behaviour × reputation showed a credible effect (*b* = −20.16, SD = 1.34, 95% CI [−22.79, −17.50]). The effect of the donor’s behaviour on their reputation varied depending on the recipient’s reputation. When the donor chose to cooperate, they were evaluated more positively when the recipient had a good reputation compared to when the recipient had a bad reputation (*b* = 4.08, SD = 0.96, 95% CI [2.21, 5.95]). Conversely, when the donor defected, they were evaluated more negatively if the recipient had a good reputation compared to a bad one (*b* = −16.08, SD = 0.10, 95% CI [−18.06, −14.08]). These results suggest that second-order information (i.e., recipient’s reputation) readily influenced how participants judged donors (see Fig. 2). These results are largely consistent with those on other impression ratings. In addition, the coefficient for group (reference: in-group) showed a credible negative effect on the donor’s reputation (*b* = −3.96, SD = 0.98, 95% CI [−5.88, −2.05]), indicating that donors were evaluated more positively when both the donor and recipient belonged to the in-group compared to the out-group. Therefore, participants appeared to generally evaluate donors more positively when both the donor and the recipient belonged to their in-group.

### 3.2. Willingness to donate

As an exploratory analysis, we examined the willingness to donate, as this variable has been used as a key dependent variable in the relevant literature (see Appendix). Detailed results on impression measurements are also provided in the online supplementary material. Following the equations used in the reputation analysis, we constructed the same set of dummy variables. The outcome variable was binary, whether participants chose to donate or not donate to the donor. We estimated the contrast parameters 
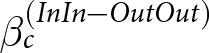
and 
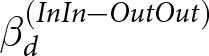
using brms. The estimate of 
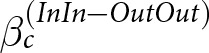
 was not credibly different from zero (*b* = 0.51, SD = 0.30, 95% CI [−0.07,1.11]), nor was the estimate of 
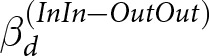
 (*b* = − 0.10, SD = 0.22, 95% CI [−0.52, 0.33]). When the donor cooperated or defected, the difference in willingness to donate based on second-order information (i.e., whether the recipient had a good or bad reputation) did not differ between the group membership of the donor and recipient.

**Figure 5.**
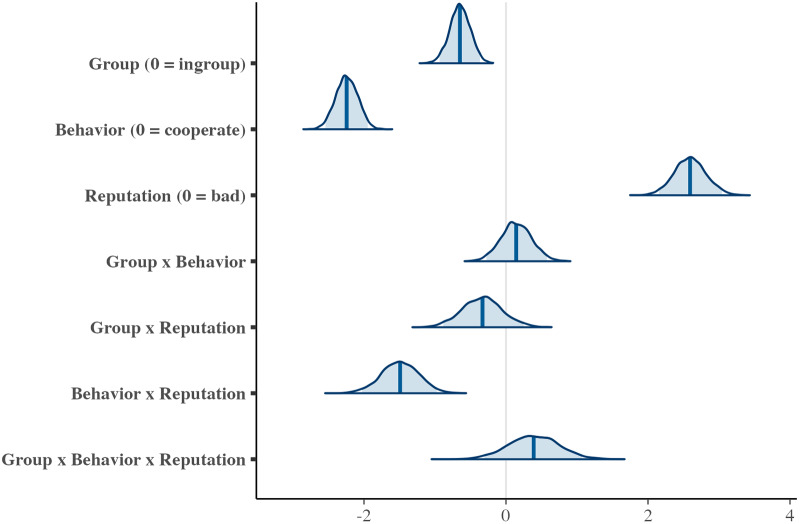
Estimated result of the willingness for donation.

*Note*: This figure shows posterior distributions of the estimated parameters. The solid vertical line represents the posterior mean, and the shaded blue areas indicate the 95% Bayesian credible intervals.

Following reputation, we conducted a 2 (donor and recipient’s group: in-group vs. out-group) × 2 (donor’s behaviour: cooperate vs. defect) × 2 (recipient’s reputation: good vs. bad) logistic regression model analysis with a random intercept (participant’s ID) on the willingness to donate to the donor (donated or did not donate, see Fig. 5). Figure 5 shows posterior distributions of the estimated parameters.

The interaction effect of behaviour × reputation showed a credible effect (*b* = −1.49, SD = 0.26, CI [−2.03, −0.98]), suggesting that willingness to donate depended on recipient reputation regardless of whether the donor cooperated or defected. Participants were more willing to donate to donors who cooperated with a recipient with a good reputation than to one with a bad reputation (*b* = 2.59, SD = 0.23, CI [2.16, 3.04]). On the other hand, they were more willing to donate to donors who defected against a recipient with a good reputation than that with a bad reputation (*b* = 1.10, SD = 0.14, CI [0.81, 1.39]), inconsistently with the social norms identified to lead to the evolution of cooperation (see Fig. 5). The coefficient of group (reference: in-group) showed a credible effect (*b* = − 0.65, SD = 0.15, 95% CI [−0.94, −0.37]), which indicates that the willingness credible effect (*b* = −0.65, SD = 0.15, 95% CI [−0.94, −0.37]), which indicates that the willingness for donation was higher when both the donor and the recipient belonged to the in-group compared to when they both belonged to the out-group.

## 4. Discussion

Drawing upon GBIR, we tested the hypothesis that individuals are more sensitive to second-order information (i.e., whether a recipient has a good or a bad reputation) when both the donor and the recipient belong to their group, compared to when they both belong to the other group. The hypothesis was supported when the donor cooperated; we found that when the donor cooperated, the difference in donor reputation based on second-order information (i.e., whether the recipient had a good or bad reputation) was larger when evaluating in-group donor–recipient pairs than when cross-group pairs or out-group pairs. Nevertheless, when the donor defected, we did not find evidence that group membership influenced the sensitivity to second-order information. In addition, in line with previous studies (e.g., Bolton et al., 2005; Mashima & Takahashi, 2008), our findings indicate that second-order information influences both participants’ impressions of the donor and their willingness to donate, regardless of whether the first-order information was cooperation or defection.

Our study was motivated by Milinski et al.’s (2001) argument that it is cognitively too costly to use second-order information. While we obtained partial support for our hypothesis, our results do not strongly suggest that individuals use second-order information predominantly within the group boundary as a way of minimising the overall cognitive load. Rather, our study suggests that individuals always refer to it to assign reputation, but their evaluation is more strongly influenced by second-order information when evaluating within-group interactions.

To understand why participants in our study, but not those in Milinski et al.’s (2001) study, used second-order information, it is worth noting the methodological difference that might have affected cognitive load and memory capacity among participants. Milinski et al. (2001) conducted a behavioural economic game study in which participants themselves played economic games, whereas participants in our study only evaluated a person playing an economic game as third parties. Therefore, those in Milinski et al. (2001) indeed had more information to process and remember to complete the study, and it is likely that they were more cognitively taxed than those in our study, allowing participants in our study to pay attention to second-order information. While we do not have a sound basis to attribute our results and the difference between ours and those reported in Milinski et al. (2001) to the difference in cognitive load, it would be a promising future avenue to directly test the effect of cognitive load and the use of second-order information in reputation assignment.

In our study, individuals used second-order information to assign a reputation to others, regardless of whether an evaluation target had cooperated or defected. Methodologically, our experiment was close to Study 3 of Yamamoto et al. (2020), and our results were largely consistent with theirs; participants generally rated donors who cooperated either as good, whether or not their recipient had a good reputation. In addition, both studies found that defection to a bad recipient is not judged as completely bad but rather as neutral, whereas defection against a good recipient was judged as bad. While Yamamoto et al. (2020) and our study together offer mixed evidence as to the use of second-order information when judging donors who cooperated before, the overall social norms individuals used are similar across the two studies. The fact that two independent studies using the same paradigm arrived at similar conclusions marks an important step towards establishing more reliable and generalizable insights into the social norms individuals use.

Regarding willingness to cooperate, unexpectedly, we found that participants were more willing to donate when the donor defected against a good recipient rather than a bad recipient, which is inconsistent with our findings on reputation judgment. This finding cannot be readily explained by the logic of indirect reciprocity. One conjecture is that cooperative donors in our study appeared too cooperative because participants were told that a donor cooperated in the past five games. Kawamura and Kusumi (2020) found that Japanese individuals liked extremely altruistic individuals less than modestly altruistic individuals. Our findings may reflect the moral conflict, suggesting that while Japanese participants acknowledge that five cooperative decisions should earn a positive reputation, they display dislike against extremely altruistic individuals (Parks & Stone, 2010). Kawamura and Kusumi (2020) found that extreme altruism was judged more unfavourably in cultures with low tolerance for social norm deviations (Japan) than in those with high tolerance (the US). Thus, this pattern was pronounced more among Japanese than Americans – the inconsistency between willingness to donate and reputation might be smaller with participants from other cultural backgrounds. That being said, at present, we do not have a clear explanation for this finding and the discrepancy between reputation evaluation and willingness to cooperate, and further research, ideally using fully incentivised cooperation measurement rather than willingness, should replicate this finding and investigate psychological mechanisms driving this.

Our study provides an important theoretical implication for GBIR. As we discussed, previous studies have gained mixed empirical evidence for the reputation management hypothesis. While some studies supported the hypothesis (Kajiwara et al., 2022; Mifune et al., 2010; Mifune & Yamagishi, 2015), other studies failed to support it by suggesting that reputational concern promotes both in-group and out-group cooperation (Romano et al., 2017) and that individuals should be worried about their reputation regardless of whether they interact with in-group or out-group members (Imada, Mifune & Zibell, 2024b). In other words, these studies failed to support the reputation management account regarding the effect of reputational concern and the sharing of reputation information. Despite this, our study suggests that group membership influenced how much individuals weigh second-order information when assigning a reputation. Specifically, in line with GBIR, second-order information influenced reputation assignment when evaluating within-group interactions – when at least one of the parties (donor or recipient) is from the out-group, the influence of second-order information wanes. We argue that it is imperative to revisit the reputation management hypothesis with a holistic approach, simultaneously investigating different aspects of indirect reciprocity: social norms/reputation assignment, gossip/reputation dissemination, and cooperation-promoting effects of reputation.

Lastly, we would like to note that by design, our study stripped away many features that characterise real-world intergroup relations, such as historical feuds, stereotypes, and group norms. Our goal in adopting the minimal group paradigm was to examine the role of group membership per se and get insights into what individuals infer from group memberships and what group membership has evolved to signal. The present findings from this context-free set-up do not necessarily suggest that we would observe the same pattern in natural intergroup contexts. The role of group membership in guiding evaluations of in-group and out-group members could be reduced or taken over in more ecologically rich settings, where evaluations are embedded within histories of intergroup relations and group-specific normative expectations. To better understand how people assign reputations in everyday life within and across group boundaries, it is sensible to study social norms in a wide range of intergroup contexts and investigate how group membership and intergroup context-specific factors together shape social norms.

## Data Availability

Data, material, and analysis code are available at https://osf.io/mkh57/overview.

## Research transparency and reproducibility interest

Data, material, and analysis code are available at https://osf.io/mkh57/overview.

## Appendix: Summary of Previous Studies

**Table:** The summary of previous studies

		Method				Result			
Article	Overview	Experimental design	Number of participants	Key experimental set up	Dependent variable	The use of first-order information	The use of second-order information Donor’s behaviour: C	The use of second-order information Donor’s behaviour: D	Details
Milinski et al. (2001).	This study conducted a behavioural experiment to investigate whether humans rely solely on first-order information (image scoring) or also use second-order information (standing) when deciding whether to cooperate with others.	Image scoring (little information: first-order information) vs. Standing (much information: second-order information)	161	This incentivised behavioural experiment involved repeated interactions in which participants made real decisions about whether to cooperate with each other. In each round, they were assigned the role of either donor or recipient and decided whether to donate points to others at a personal cost. Their decisions were visible to the group, enabling analysis based on image scoring and standing strategies. The experiment alternated between a “low-information” condition (only the donor’s past behaviour was visible) and a “high-information” condition (both the donor’s and recipient’s past behaviours were visible). One participant in each group was secretly instructed to always choose “NO” (the NO player). The game lasted 16 rounds.	Cooperation rate: Probability that donors who cooperated or defected with a defector are themselves defected against	◯	×	×	There was no significant difference in the probability that donors who interacted with the NO player (i.e., defector) were defected against between the two conditions (first-order information vs. second-order information). This suggests that participants’ behaviour was more consistent with an image-scoring strategy than with a standing strategy.
Bolton et al. (2005)	This study conducted a behavioural experiment to investigate what kind of information (none, first-order information, second-order information) promote cooperation.	Cost of cooperation (low vs. high) × Information (no information vs. first-order information vs. second-order information)	192	This incentivized behavioural experiment involved repeated interactions in which participants made real decisions about whether to cooperate with each other. In each of the 14 rounds, participants were assigned as either a donor or a recipient. The donor decided whether to give money to the recipient at a personal cost or to keep it. The experiment included three conditions: in the no-information condition, no information was available; in the first-order information condition, only the recipient’s past behaviour was shown; and in the second-order information condition, both the recipient’s and their previous recipients’ behaviours were displayed.	Cooperation rate: how often participants cooperated with recipients when assigned as donors	◯	◯	◯	Cooperation rates were highest when second-order information was available. A closer examination revealed that donors who cooperated with good recipients were more likely to be cooperated with than those who cooperated with bad recipients. Similarly, among those who defected, donors who defected against bad recipients were more likely to receive cooperation than those who defected against good recipients.
Mashima and Takahashi (2008)	This study conducted a vignette experiment to investigate evaluations of a donor when first-order information and second-order information were provided.	First-order information (a donor’s behaviour toward a recipient: cooperate vs. defect) × Second-order information (a recipient’s reputation: good vs. bad)	282	This study conducted a vignette experiment involving six scenarios in which participants, as third-party observers, evaluated a donor based on first-order and second-order information. They were also asked to imagine whether they would cooperate with the donor. There were four donor types: one who cooperated with a good recipient, one who defected against a good recipient, one who cooperated with a bad recipient, and one who defected against a bad recipient.	Evaluation of donors (generousness, social appropriateness, social order, expectation of positive treatment by others) Willingness to cooperate with donor	◯	◯	◯	Participants evaluated donors based not only on first-order but also on second-order information. Defection against bad recipients was evaluated more positively than defection against good recipients. Additionally, while cooperation with bad recipients was perceived as more generous, cooperation with good recipients was rated higher in terms of social appropriateness, social order, expectations of positive treatment by others, and willingness to cooperate.
Ule et al. (2009)	This study conducted a behavioural experiment to investigate the role of first-order and second-order information in indirect punishment and rewarding. It focused on how individuals use these types of information to make decisions about helping (i.e., cooperate), passing (i.e., defect), and punishing and divided them to strategies.	Symbolic punishment (SP) vs. Harmful punishment (HP) The punishment is harmful in HP condition but only symbolic in SP condition.	140	This incentivised behavioural experiment involved repeated interactions in which participants made real decisions about how to act toward each other. Donors were paired with recipients and chose to help (cooperate, incurring a personal cost to benefit the recipient), pass (defect), or punish (incurring a cost to harm the recipient). Donors were given first-order information (the recipient’s recent actions) and could access second-order information (what the recipient previously observed) at a small cost. Two punishment types were used: harmful punishment and symbolic punishment. The game lasted for 100 rounds.	The percentages of participants’ strategy	◯	△		Participants who conditionally cooperated relied more heavily on image scoring than on standing. Specifically, in the SP condition, 50.0% followed an image-scoring strategy and 25.0% followed a standing strategy. In the HP condition, these figures were 39.5% and 17.1%, respectively. Overall, twice as many participants relied solely on first-order information, indicating a general preference for image scoring over standing, despite individual differences.
Swakman et al. (2016)	This study conducted a behavioural experiment to examine whether people condition their cooperation on second-order information. Participants decided whether to cooperate with a recipient after viewing first-order information. There were three conditions regarding second-order information: Control (not available), Free (available at no cost), and Costly (available at a cost).	The ease of access to second-order information (unavailable vs. free vs. costly)	160	This incentivized behavioural experiment involved repeated interactions in which participants made real decisions about whether to cooperate with each other. Participants were divided into groups of 10 and randomly assigned as either a donor or a recipient in each round. Donors chose whether to cooperate or defect with an anonymous recipient. If a donor cooperated, the recipient’s earnings increased by 250 points, while the donor’s earnings decreased by 200 points; if the donor defected, neither participant’s earnings changed. The game lasted for 100 rounds. All participants had access to first-order information (the recipient’s past decisions), but the ease of access to second-order information varied across three conditions: control (unavailable), free (freely accessible), and costly (available at a cost).	The percentages of participants’ strategy Whether to access second-order information	◯	×	◯	This study identified three key insights into reputation-based cooperation. First, many participants actively sought second-order information, even when it required a cost, indicating a strong interest in the motivations behind others’ decisions. Second, participants paid particular attention to second-order information about defection, and justified defection (i.e., refusing to cooperate with defectors) was often rewarded. Third, there was substantial individual variation in strategy use: while approximately 40% relied solely on first-order information (in both free and costly conditions), others integrated second-order information into their decisions (about 30% in the free condition and 10% in the costly condition).
Okada et al. (2018).	This study conducted a behavioural experiment to investigate selective inattention in reputation-based cooperation. It examined whether individuals deliberately use or ignore first-order information and second-order information when deciding whether to cooperate.	-	152	This incentivized behavioural experiment consisted of 50 rounds in which participants, given 2000 points, decided whether to cooperate or defect with an anonymous recipient controlled by a computer program. Cooperation increased the recipient’s earnings by 300 points and reduced the participant’s by 100, while defection left both unchanged. Participants were also assigned a virtual donor. Before each decision, they could freely access first-order information (the recipient’s past actions) and second-order information (the behaviour of the recipient’s previous partners). Although the interactions were simulated, participants were told they were interacting with real people. Participant’s outcomes were determined by the program.	Information disclosure behaviour Determinants of cooperation (first-order information, second-order information)	◯	×	◯	Participants tended to disclose first-order information more frequently than second-order information. When both types were disclosed, decisions were still primarily based on first-order information. Specifically, participants were likely to cooperate when recipients’ previous actions were cooperative (‘C’), regardless of second-order information. In contrast, when recipients previously defected (‘D’), participants were more inclined to consult second-order information to judge whether the defection was justified. However, those who first disclosed second-order information did not necessarily use it in their decisions, often defaulting to first-order cues or other factor
Yamamoto et al. (2020)	This study conducted a vignette experiment to investigate the distribution of donor’s evaluations when first-order information and second-order information were provided.	First-order information (a donor’s behaviour toward a recipient): cooperate vs. defect) × Second-order information (a recipient’s reputation: good vs. bad)	200	This study conducted a vignette experiment involving three scenarios in which participants, as third-party observers, evaluated a donor based on first-order and second-order information. There were four types of donors: one who cooperated with a good recipient, one who defected against a good recipient, one who cooperated with a bad recipient, and one who defected against a bad recipient.	Evaluation of donors	◯	×	◯	An analysis of donor evaluations revealed consistent patterns. Cooperation with both good and bad recipients was generally rated positively, whereas defection against good recipients was rated negatively. In contrast, defection against bad recipients elicited a neutral evaluation, with responses centred around neither good nor bad, indicating ambiguity in moral judgement for that case.
